# Integrating Spherical Panoramas and Maps for Visualization of Cultural Heritage Objects Using Virtual Reality Technology

**DOI:** 10.3390/s17040829

**Published:** 2017-04-11

**Authors:** Mila Koeva, Mila Luleva, Plamen Maldjanski

**Affiliations:** 1 Faculty of Geo-information Science and Earth Observation, University of Twente, Hengelosestraat 99, 7514 AE Enschede, The Netherlands; 2 SoilCares Research BV, 6709 PA Wageningen, The Netherlands; mila.luleva@soilcaresresearch.com; 3 University of Architecture Civil Engineering and Geodesy, Sofia 1164, Bulgaria; maldjanp_fgs@uacg.bg

**Keywords:** virtual reality, 3D modelling, photogrammetry, visualization, panoramic images, integration, interactive visualization, cultural heritage

## Abstract

Development and virtual representation of 3D models of Cultural Heritage (CH) objects has triggered great interest over the past decade. The main reason for this is the rapid development in the fields of photogrammetry and remote sensing, laser scanning, and computer vision. The advantages of using 3D models for restoration, preservation, and documentation of valuable historical and architectural objects have been numerously demonstrated by scientists in the field. Moreover, 3D model visualization in virtual reality has been recognized as an efficient, fast, and easy way of representing a variety of objects worldwide for present-day users, who have stringent requirements and high expectations. However, the main focus of recent research is the visual, geometric, and textural characteristics of a single concrete object, while integration of large numbers of models with additional information—such as historical overview, detailed description, and location—are missing. Such integrated information can be beneficial, not only for tourism but also for accurate documentation. For that reason, we demonstrate in this paper an integration of high-resolution spherical panoramas, a variety of maps, GNSS, sound, video, and text information for representation of numerous cultural heritage objects. These are then displayed in a web-based portal with an intuitive interface. The users have the opportunity to choose freely from the provided information, and decide for themselves what is interesting to visit. Based on the created web application, we provide suggestions and guidelines for similar studies. We selected objects, which are located in Bulgaria—a country with thousands of years of history and cultural heritage dating back to ancient civilizations. The methods used in this research are applicable for any type of spherical or cylindrical images and can be easily followed and applied in various domains. After a visual and metric assessment of the panoramas and the evaluation of the web-portal, we conclude that this novel approach is a very effective, fast, informative, and accurate way to present, disseminate, and document cultural heritage objects.

## 1. Introduction

In recent years, advances in the field of photogrammetry and remote sensing, laser scanning, computer graphics, robotics, surveying, and internet-related technologies have opened up new opportunities for visualization and proper documentation of cultural heritage (natural, cultural, or mixed) [1]. These new technologies help to preserve the memory of historic buildings, archaeological sites, and landscapes [1,2]. Web-based Virtual Reality (VR) environments are an easy way to represent and disseminate Cultural Heritage (CH) among a variety of people. It supports a country’s economic growth by stimulating cultural tourism. Therefore, preservation and visualization of such places of interest have always been a serious challenge for specialists in the field of cultural heritage. There are a number of problems that specialists can face during the process of 3D modelling for cultural heritage representation. These include difficulties in selecting the technology for image acquisition and post-processing, defining the proper workflow in order to acquire a high visual and metric quality of the final products, integrating other types of data (including archives), and web-based visualization. As in other domains, one of the major issues is how to manage, process, and disseminate variety of data via the internet in an application that is easy to use. This data requires storage space, powerful processing software, and hardware and experienced professionals. To find the balance between quality and speed (speed of rendering and speed of downloading data) is quite challenging. Nowadays, an impressive evolution has been observed in 3D model creation, not only for existing objects, buildings, or complete cities, but also for virtual reconstruction of destroyed or damaged objects. Resent research in the field proved the contribution of 3D modelling and visualization for restoration, preservation, conservation, and documentation of CH [3,4,5,6], web-based computer-aided restoration [7], and multimedia museums [8].

Various acquisition techniques have been used for 3D model creation both aerial and terrestrial. Low-cost solutions with up-to-date fit-for-purpose technologies have also been found suitable for this task [9,10,11]. Unmanned Aerial Vehicles (UAV) independently and in combination with Terrestrial Laser Scanning (TLS), have been considered as very suitable acquisition techniques in terms of accuracy, resolution, and coverage for cultural heritage [12,13,14]. 3D modelling of historical objects as well as image-based modelling using laser scanning technology, aiming in automatization, have shown great potential [15,16,17,18,19,20,21].

Naturally, the selection of the most suitable and effective technology is strongly related to the specific requirements of each project, its budget and the experience of the users. 3D modelling and visualization for objects with high level of complexity, supported with additional information and map representation, is still a challenging topic for most researchers in the field. A variety of combinations from the available acquisition techniques have been investigated by researchers [22,23,24,25,26,27,28,29]. More precisely, the main focus has been on metric capabilities with high-resolution rotating cameras [30,31,32,33,34,35]. Due to the large field-of-view limiting the number of acquisitions and the high-resolution content, photorealistic modelling, the use of spherical images for spherical photogrammetry for photorealistic modeling, has recently received increasing attention. In addition, researchers working on representation of architectural and archaeological objects have shown great interest in using spherical photogrammetry for photorealistic modelling. The main reason for this is the low cost of the equipment used for acquisition of spherical images [36,37,38,39,40]. These researchers have shown that high-resolution accurate data obtained using such technology are extremely beneficial for the creation of 3D models of cultural heritage objects.

Therefore, the technological development in the fields of photogrammetry, laser scanning, and computer vision nowadays is providing opportunities for web-based Virtual Reality (VR) visualization. It is a very efficient, fast, and easy way to represent architectural and archaeological objects worldwide for variety of users [41]. Virtual reality has extensively been used recently in various applications such as medicine [42], architecture and environmental planning [43], mechanical and aerospace engineering [44], and education [45]. The evolution of virtual data representation possibilities such as Virtual Reality Modelling language (VRML) web-based applications, together with the easy and widely available internet access, are becoming a very suitable solutions for dissemination of cultural heritage [46,47].

Following the developments in the field, scientists in Bulgaria have also put a lot of effort in to preserving national cultural heritage and keeping inherited traditions from the past. In 2008, as an example of the development of virtual reality representations of heritage in the region, Sofia’s Virtual Museum was developed. Other interesting results have been presented by the Bulgarian Academy of Science at the First International Workshop on Virtual Archaeology, Museums & Cultural Tourism in Greece (2013). Miglena Vasileva presented several examples of 3D reconstructions, including the Grand Palace with the throne room in Pliska and the Kings complex in Preslav. To achieve a high degree of realism, numerous computer graphics techniques with a combination of integrated audio and video effects were shown. Other examples of 3D have been reported by Maldzhanski and Koeva [48,49,50].

Many researchers shared the need of representation of cultural heritage using virtual and augmented reality. The first tests using virtual and augmented reality were reported in [51,52,53,54]. The advantages were shared by Reffat in 2013 [55] and, later on, the problems related with standardization of the user interface were discussed in the computer vision community [56]. The usage of spherical photogrammetry for these purposes was also extensively researched nationally (in Bulgaria) and internationally [57,58]. However, current research in the field focuses mostly on the photogrammetric principles and technologies for a single model creation and object restoration [59] or a museum including several indoor objects using simplified map representation only for basic orientation, which are presented through a “virtual tour”. An example of this is the Louvre Virtual Museum [60] where spherical panoramic images and frame pictures are integrated into a portal. However, the resolution is low and there is no map representation. Our work differs from these studies and improves on the existing methods. The study provides an innovative way of bringing together diverse types of data for visualization of cultural heritage, which requires storage space, software, hardware, and expertise to be processed and integrated into a fast and high quality web application.

In the current study, we provide a methodology for efficient acquisition, use, management, integration, and dissemination of various types of data for effective visualization and documentation of cultural heritage objects. More specifically, we investigate the processing and usage of high-resolution spherical images for visualization of numerous objects into a fast and easily accessible web application. This is also an innovative approach, which has not been reported by the scientific community in the field to date.

We performed a thorough analysis and review of studies in the field of visualization for cultural heritage. Furthermore, based on this review, we carried out an evaluation of the available techniques in order to select the most appropriate one. Therefore, the main scientific contribution of the current research is the use of remote sensing technology for this application including the successful integration of: (i) numerous high-resolution spherical panoramic images (indoors and outdoors), (ii) a variety of interactive maps, and (iii) multimedia information. This information is processed, organized, linked, and integrated into a web-based portal with an intuitive interface for exploration and documentation of cultural heritage objects.

Efficient data integration, management, documentation, and representation of cultural heritage and landscapes today are a strategic priority not only to assure cultural treasures but to exploit them as valuable economic assets. Furthermore, we provide information, suggestions, and general guidelines for the processing and usage of spherical or cylindrical images, combined with other data for web representation. These can be followed and applied in various scientific domains such as medicine, architecture, education, environmental planning, etc.

Our research presents a tested and applied methodology using remotely sensed data acquired in a cheap, fast, and accurate way from a terrestrial platform, efficiently combined with map representation, GNSS data, and other multimedia information. We believe this makes it valuable for the scientific community and introduces a novelty to the field.

## 2. Materials and Methods

There are two types of panoramic images: cylindrical and spherical. Both can be acquired with linear array and rotating panoramic cameras, suitable for providing high-resolution images. There are two methods for handling such images. The first method is based on 3D scene reconstruction, for example, through space intersection, from multiple spherical panoramic images generated by stitching frame images [61]. This is the process of aligning and projecting the images for the creation of multi-image photos. The second method is to acquire overlapping images from a single point. Such an acquisition is possible when using a frame-CCD camera rotated around its perspective center, mounted on a dedicated tripod. During the image acquisition, the projection center and the focal length remain constant. Once created, the spherical images are mapped using equirectangular projection [62]. The relationship between a point on an image and a point on the object can be expressed by two collinearity equations [63]. The images are stitched together and are projected on a virtual sphere whose radius is equal to the focal length of the camera [64]. Nowadays, there are numerous software programs used for automatic mosaicking of images (stitching).

The primary goal of this research was to investigate how appropriate the spherical panoramas are for integration with other types of data in a virtual reality environment. This is why extensive metric information was not considered necessary. However, we made an investigation on the suitability of the acquired panoramas for metric purposes in case the user would like to perform measurements using the spherical panoramas. Distance measurements, using a tape, were taken only for several selected objects during the image acquisition period. Ground Control Points (GCP) were not measured on the objects. Therefore, since we had only one spherical image per object and no stereoscopy was possible, we followed the methodology used and described by Fangi [37,38,39]. The method is called “monoplotting” and is suitable when the surface of the object is known. Then as it was described by Fangi it is possible to intersect this surface with the projective rays coming only from one panorama.

We did not measure GCPs since this was out of the scope of our current research. For several selected objects during the image acquisition period, distance measurements with a tape were taken, in order to evaluate the metric qualities of the created panoramas. We measured distances between easily distinguishable points on the objects which can be easily found and measured on the images. The results from our tests, using monoplotting, which we also compared with results obtained from other researchers in the field, can be taken as an additional information for the users who would like to use panoramas for measurements.

In this research, we selected Tourweaver^®^ (Easypano Holding Inc., Shanghai, China) [65] to develop a virtual representation. It allows the integration of numerous panoramas into a web-portal.

### 2.1. Study Area

We selected historical objects located in four municipalities in the Central-Northern part of Bulgaria—Lovech, Troyan, Letniza, Aprilci. Some of the oldest towns in Bulgaria are located in this region including Lovech and Troyan. Their first inhabitants were Tracians, whose traces date back to the third century BC. Over the centuries, the region became famous for its unique archaeological and architectural heritage, applied arts and crafts, ethnography, and folklore. Therefore, with the development in information technology, the questions surrounding documentation, preservation, and popularization of the valuable cultural heritage have undoubtedly been raised [66].

### 2.2. Object Selection and Location

**Figure 1:**
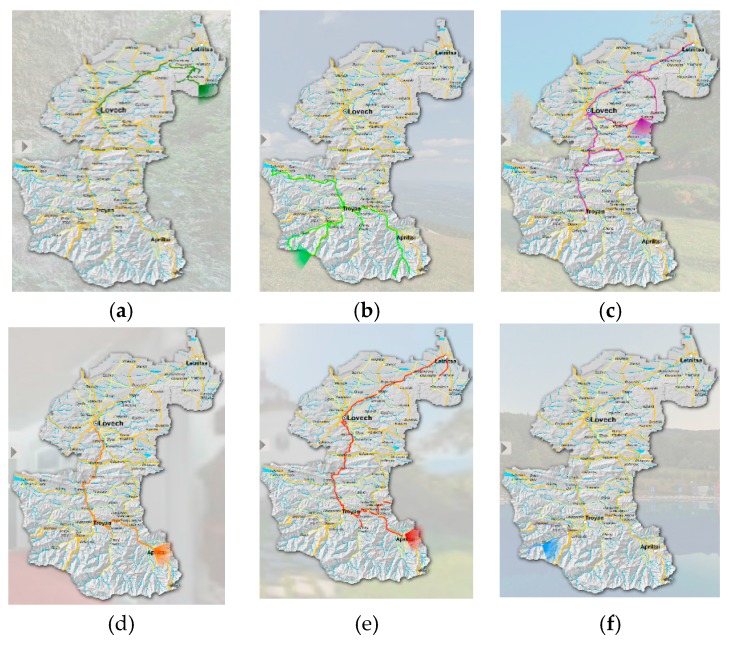
Map of the region representing the four municipalities and maps with the distribution of 50 objects into six routes. (**a**,**b**) natural (with caves, waterfalls, eco paths etc.), (**c**) cultural (galleries, museums, historical, archaeological, and architectural monuments), (**d**) traditional (museums for applied arts and crafts), (**e**) religious (churches and monasteries), and (**f**) recreation (mineral water resources).

For the purpose of the study, 50 objects were chosen, as they are considered interesting to be visited by potential tourists. These objects were then grouped according to themes and located along six touristic routes. These are: (1) and (2) natural (with caves, waterfalls, eco paths, etc.), (3) cultural (galleries, museums, historical, archaeological, and architectural monuments), (4) traditional (museums for applied arts and crafts), (5) religious (churches and monasteries), and (6) recreation (mineral water resources). Each of these routes was visualized using ArcGIS software package, creating a map with 3D survey representation (Figure 1). Coordinates of the objects on the routes were determined using hand-held Garmin GNSS.

### 2.3. Image Acquisition and Post Processing

For some of the objects, such as churches and monasteries, signed permission for their acquisition was needed which was obtained with the help of municipalities. Technical equipment used for acquiring spherical images was Canon EOS 1Ds Mark 3 (5616 × 3744) with Sigma 15 mm, f/2.8L lenses mounted on a Manfrotto 808RC4 with spherical tripod head Nodal Ninja 5R with D16 rotator. This spherical head has a special adjustment that allows it to be rotated on a tripod over the “pupil” to eliminate any vertical parallax (shift of the foreground objects relative to the background ones). The images were taken using f9-stop aperture in order to use the maximum advantages of the lenses.

The camera was placed on a tripod Manfrotto 808RC4, and the height of the set-up was set to 1.5 m. The camera was then remotely controlled from a minimum distance of 5 m. The camera rotated automatically and took images at regular intervals of 60°. The distance between the tripod and the objects at the time of capturing the panoramas, was 2–8 m.

The original raw images were converted with specialized software Capture One7 [67], while the stitching for panorama creation was done using High Dynamic Range (HDR) Expose 3 and SNS-HDR Pro. This stitching software was selected because it offers automatic removal of the shadow created by the tripod and automatic equalization of brightness and contrast differences, which undoubtedly exist in every mosaicking process. This system is definitely easy for use and satisfies the requirements for a flexible and seamless stitching process. In cases where smooth visualization or correction of disturbances was required, Adobe Photoshop CS6 [68] was used.

### 2.4. Panorama Creation

**Figure 2:**
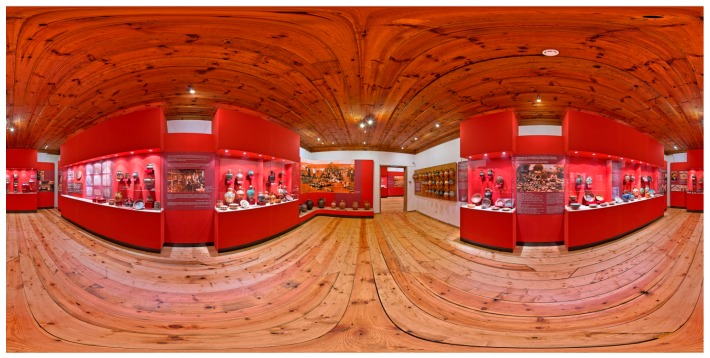
Example of spherical panorama. Museum of crafts and applied arts in Troyan.

**Figure 3:**
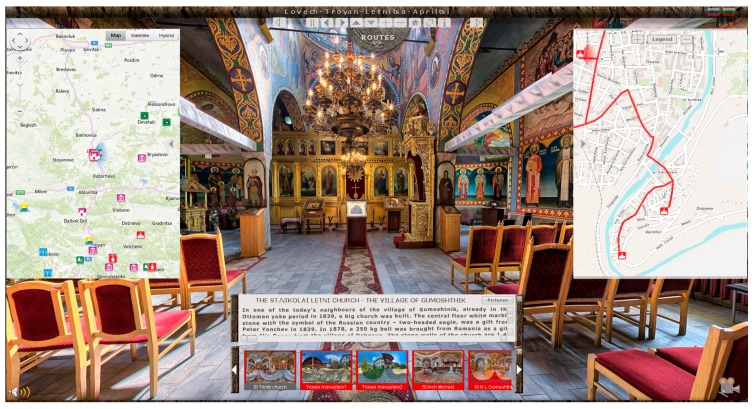
Map appearance over the spherical panorama of the Holy Trinity Orthodox Church in Lovech.

For the creation of 50 (exterior and interior) panoramas, a total of 3200 images were captured and stitched using Pano2VR^4^ 64 bit and PTGui [69]. The final panoramas were saved into lower resolution (see example in Figure 2). The downsampling of the images had to be performed because of technical limitations of the web-server of the municipalities (Lovech, Troyan, Letniza, Aprilci) and to allow users a fast and smooth visualization experience of the cultural heritage sites. We tested different downsampling ratios in order to find the balance between image resolution and computational efficiency in terms of rendering and downloading speed. The aim was to have a fast, easy, and smooth visualization everywhere in the world with less possible technical requirements. In this way, we allowed also users with limited internet bandwidth connection to access and smoothly browse the web portal. For the portal, panoramas were divided into themes and visualized in “thumbnails” at the bottom of the screen (Figure 3) in different colors just like the routes on the maps.

### 2.5. Additional Input Data for the Web-Portal

High- resolution frame images were additionally captured with Canon EOS 5D Mark 2 (5616 × 3744) with lenses EF 24-70, f/2.8L USM; EF 70-200, f/2.8L USM telephoto extender Extender EF 2× II mounted on a Slik tripod. The different type of lenses were used only for the normal frame images. For the spherical panoramas, we used only Sigma 15 mm, f/2.8L. Therefore, an open source tool was integrated into the portal for their visualization. Team of professional actors were asked to record their experience while visiting all of the selected cultural objects in a video format. They recorded historical information and took interviews with the local people. The video, which was delivering all the information for needed tourists, was uploaded into YouTube and the link was imported into the web-portal (see Figure 3 bottom right). Additionally, while navigating in the portal, the sound of nature and birds was recorded and integrated with the option to be switched off and on depending on the users’ wish (see Figure 3 bottom left).

To integrate the obtained spherical panoramas with interactive maps, video, sound, and text information, Tour Weaver software was selected. Since user interface is not standardized, VRML browsers complying with ISO standard differ. Since the selected software is developed to be applied in different domains, there was no suitable interface in the offered ones for the current task. Therefore, we designed every button that had to be visualized in the viewer in CorelDraw, in different sizes and colors, depending on its function (active or passive, switched on/off etc.).

**Figure 4:**
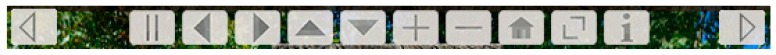
Screenshot from the web-portal.

Several versions of the overall design were developed to ensure that users’ needs are met. Navigation tools were positioned at the top of the screen to assist users. Buttons were made to adjust the speed of the visualization, and to allow going back and forward to the next panorama, and to zoom in and out. In addition, we inserted a home button and a switch-to-full-screen and information button. (Figure 4)

A switch is designed to be used by the users in case they would like to select an alternative route. The users are also able to select between specific objects along the selected routes through two arrows, which we positioned right and left in the screen. We made an information button, marked with (i), which is enabled when a specific object is selected. There is also a picture button, which visualizes frame images of the selected object. In addition, we integrated a Bing map with Satellite and Hybrid options. Moreover, using ArcGIS software, we additionally created different map representations for the central prats of the cities with more details (Figure 3). On these maps, the routes were separately visualized with a unique color. We performed a number of tests concerning map representation, until we were able to obtain smooth and fast web visualization.

The additional rotating radar function was integrated in the portal in order to help the user with orientation. This function is to be enabled and used simultaneously with selecting an object. To solve the problem with the large size of the data, the panoramas were resampled from the original full resolution before being imported in the web-portal.

The web-portal was tested before publishing from five potential users. We asked them to share their observations, recommendations, and difficulties while navigating, locating, and using it. This gave us precious feedback which was used to improve its functionality and to make it more user-friendly.

### 2.6. General Guidelines

In the current research the procedures described in the subsections above can be used as general guidelines for similar studies in different domains. To underline the most important steps among them, we can summarize them in the following baseline indications:

- Selection of a study area and objects for representation
- Acquisition of permissions for objects acquisition
- Selection of the acquisition technology
- Image acquisition, image post-processing, and image refinement
- Panorama stitching and rendering (if spherical or cylindrical panoramas are used)
- Software selection for virtual representation
- Map creation and adjustment for their visualization in different resolutions and browsers
- Selection of additional information for multimedia representation (GNSS objects coordinates, frame images, video, sound, etc.)
- Selecting and experimenting with different options for their better visualization
- Design of user friendly interface of the application
- Testing the performance of the created web (or offline working) application until approval

## 3. Results

From the website of the four municipalities, the result of the current study the web portal can be visualized and ran in all common browsers, such as Mozilla Firefox, Internet Explorer, and Google Chrome [70] (Figure 3). From the upper right corner, the user can select the preferred language from English, Bulgarian, or Russian.

To assess the obtained metric qualities of the spherical panoramas we used the method called “monoplotting” theoretically described and applied by Fangi [37,38,39,40,71]. For several selected objects during the image acquisition period, distance measurements with a tape were taken in order to evaluate the metric qualities of the created panoramas. We measured distances between easily distinguishable points (corners) on the objects which can be easily found and measured on the images. Using Erdas software, we transformed the panoramas of the selected objects and we measured in their central parts where no distortions were observed. For the objects with irregular or oval shape taken from a distance less than 5 m. In reality, after comparison of the distance measurements taken with a tape on the real objects and measure in the software the result of the difference was 0.1 m. A similar experiment was done on a panorama taken from a longer distance where we obtained a difference of 0.3 m. However, after we selected an indoor panorama of a museum with a clear linear structure and features which lie on a plane, we obtained better results at 0.01 m. For evaluation of the achieved results, we made compared our results with those obtained from other researchers using the same method. We found that Karras et al. [72] reported similar results to ours. After resampling of the images, performing radiometric corrections, and mosaic creation they calculated the RMS differences of similarity transformation between mosaic and control points in X and Y = 0.014 m and in Z = 0.005 m. The difference in their research is that the distortions and inaccuracies in their case were mainly due to the cylindrical object they were investigating.

**Figure 5:**
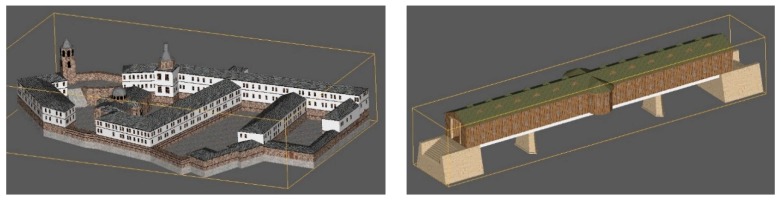
3D models of Troyan Monastery and the Covered Bridge in Lovech created in 3D StudioMax.

**Figure 6:**
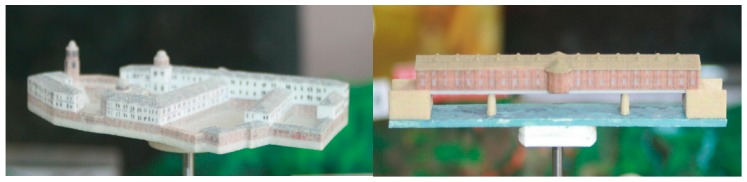
3D printed models of Troyan Monastery and the Covered Bridge in Lovech.

We also created 3D physical models of some of the cultural heritage objects. Such a method was used because, in countries such as Bulgaria, sometimes a digital way of information sharing on the internet is not enough. The 3D models were created based on the acquired frame images using 3D StudioMax software (Figure 5) and printed using 3D Systems-ProJet® 460Plus [73]. This is a full color 3D printer which operates with safe build materials, active dust control, and zero liquid waste. The models were provided to the municipalities to catch people’s attention and encourage tourists to view the portal and to visit the cultural heritage objects (Figure 6). The scale of the printed model is 1:500.

## 4. Discussion

The added value of 3D modelling and visualization has been demonstrated by many researchers [3,4,5,6]. Particularly, its importance for preserving cultural heritage is great because it gives us the opportunity to visualize spatial and temporal information for precious historical monuments and objects of interest. However, 3D modelling and visualization have proven to be challenging. This is mainly due to difficulties with data acquisition, data integration, and virtual representation. There is a great availability of sensors and platforms which is continuously developing [74]. The combinations and integration of various data, automation, and real-time processing are topics that are in continuous research and will be further developed in the future [75]. However, the use of more sophisticated technology does not always result in a user-friendly and readily-available product. This is where the method presented in this paper adds value. It is an affordable, fast, and effective method for the visualization of valuable historical and natural places in a modern and easy accessible way. The product can be accessed via a web portal, while the geographical locations can be easily downloaded onto any portable GNSS device. Assessment was done by investigating the level of similarities between virtual representation and real world [76,77,78]. It was concluded that such spherical panoramas integrated in a web-portal with additional types of information can be undoubtedly used for the interactive representation of important historical monuments, archaeological and architectural objects, and other places important for cultural preservation. Due to the high resolution of the acquired spherical panoramas the results from the visual assessment were high. Even resampling to the lower resolution, which was needed for smooth navigation in the web environment, did not lower the visual qualities of the panoramas.

Except from visual assessment, we performed metric assessment of the quality of the panoramas. Using the monoplotting method described by Fangi [37,38,39,40], the obtained metric qualities of the tested planar object in the current research was 0.01 m. Since metric investigation was not the primary goal of the research, we performed tape distance measurements of the objects in reality and compared the results with the those measured on the panorama which was transformed in Erdas software. The information of our accuracy test can be considered as additional information for users who would like to use panoramas for measurements. If there is a need of extracting detailed metric information from spherical images, then we recommend precise measurements of Ground Control Points (GCPs) on the objects to be made. The accuracy and reliability of this source of information has been proven by a number of authors [37,40,61,79,80,81]. For example, measuring control directions and control points with a reflectorless theodolite, the RMSE of the residuals obtained in the tests of Fangi were 0.027 m in planimetry and 0.009 m in height over 108 control points observed at least in three panoramic images. Alternative solutions for improving metric qualities using multiple spherical images and testing different stitching methods were also researched [82,83].

Metrically, the method of monoplotting we used in this research is suitable for planar surfaces. For the current goal of this research the achieved visual and metric qualities were fully satisfactory. For applications which require detailed representation and high metric qualities, the following methods can be used: (i) for solving additional geometrical problems as in [6,39,66,84], (ii) interactive modelling as in [82], and (iii) automatic orientation procedures as in [36].

There are three main factors that determine the quality of the integrated spherical panoramas with additional information into a web portal. These are appropriate selection of objects of interest based on user experience, accurate selection of the equipment for the data acquisition and data processing tools, and development of a user-friendly and easily accessible web-based platform or portal.

### 4.1. Selection of Objects of Interest

The objects of interests were selected in collaboration with people from each of the four municipalities. With the help of their expert knowledge, the objects of interest were grouped according to themes, and located along six officially defined touristic routes. These groups are: (1) and (2) natural (with caves, waterfalls, eco paths, etc.), (3) cultural (galleries, museums, historical, archaeological, and architectural monuments), (4) traditional (museums for applied arts and crafts), (5) religious (churches and monasteries), and (6) recreation (mineral water resources).

### 4.2. Selection of Appropriate Equipment and Data Processing Tools

Canon EOS 1Ds Mark 3 and 5D Mark 2 camera with a D16 Rotator. The advantages of the D16 Rotator is that the user has sixteen choices for the spacing of “click stops”, as the head rotates [85]. The acquired images offer the possibility for accurate and attractive visualization, while allowing integration of additional information into a web-portal. The overlap between the images depends on the distance from the camera to the object. When using “manual mode” of the f9-stop aperture, we take maximum advantage of the lenses. The exposure time was 1/250–30 s, using the principle of Zeitraffer photography, which is needed for the software that we used for the post-processing of the images.

For the visual and metric qualities of the panoramas the resolution of the raw data and the methods used for post-processing are of great importance. For the current task the visual quality was very good due to the high-resolution of the input data and the post-processing was accurately done in order to avoid any distortions.

### 4.3. Web Portal

The aim of the web-portal was to integrate different types of input information, in order to provide the users with different possibilities to make their choices. As a basis, the acquired spherical images were used; however, additionally high resolution frame images were also linked to the objects. They were visualized in a separate view done via integrating an open source tool for this purpose in the portal. They appear at a click of the button “Pictures”. Since video has been considered as one of the most powerful and efficient methods of visual communication and representation, we also decided to include video in the portal in order to achieve our goal.

In the designing of the web-portal, the most labor-and-time intensive tasks are linking the buttons and assigning the action that they have to perform together with setting the time interval. After all the settings have been selected, by clicking on a selected touristic object, the user can freely navigate in the panoramic view, view an object location on several maps representations, obtain information about its position (GPS coordinates), select from the small panorama visualization in “Thumbnails”, investigate the attached frame images, watch the video, and read the text containing historical information (Figure 3).

## 5. Conclusions

The paper presented the first virtual web-portal with an integration of numerous high-resolution spherical panoramas, a variety of maps, frame images, GPS coordinates for the touristic routes, sound, video, and text information representing the cultural heritage in Bulgaria. This result was evaluated highly and positively accepted and presented nationally (Sofia, Veliko Turnovo, Russe) and internationally (Finland, Germany, Great Britain, Russia). On a more local level, achievements were presented in the news, local magazines and newspapers and were supported by government and non-government organizations. We demonstrated an example of an operational and reproducible procedure as it is easy to use, inexpensive, and presents reliable quality.

This research concluded that the visual and metric qualities of high-resolution spherical panoramas are sufficient for many applications. For tourism, virtual navigation, documentation, and demonstration of numerous cultural objects, panoramas supported with additional information and integrated into a web-portal are a suitable solution. As a recommendation, such interactive visualizations can potentially be extended with virtual tours using avatars, perhaps even with a voice. Further improvements are related to usage of more automatic web-based visualization using interactive geo-special information.

The topic is in constant research and, especially with rapid technological development, even more accurate and detailed 3D models are expected to be used in web-based virtual reality applications.

The availability of digital data and its use for 3D modelling and visualization opens possibilities for variety of application and opportunities for analysis and interpretations. However, some misunderstandings between technical scientists and specialists in cultural heritage exist, which have to be solved in order to provide efficient documentation and preservation. The current study is a concrete example of how such a problem can be solved.
